# Obesity may impair response to ovarian stimulation. A retrospective observational study on oocyte quality

**DOI:** 10.3389/fcell.2024.1461132

**Published:** 2024-09-30

**Authors:** Irene Iavarone, Daniela Mele, Francesca Caprio, Giada Andreoli, Maria Giovanna Vastarella, Pasquale de Franciscis, Carlo Ronsini

**Affiliations:** Department of Woman, Child and General and Specialized Surgery, University of Campania “Luigi Vanvitelli”, Naples, Italy

**Keywords:** body mass index, antral follicle count, mature oocytes, obesity, ovarian reserve

## Abstract

**Background:**

Ovulatory dysfunction is more common in women with obesity. Body fat distribution is also crucial because anovulatory women have a greater waist circumference and more abdominal fat than ovulatory women of similar BMI. The primary aim of the present study is to determine whether there is a relationship between BMI and reproductive characteristics, including hormonal values, antral follicle count (AFC), endometrial assessment at transvaginal ultrasound evaluation (TVUS) during controlled ovarian stimulation (COS), and oocyte retrieval after Ovum Pick-Up (OPU).

**Methods:**

Data from a cohort of 183 patients were analyzed and divided into three groups based on weight status: normal weight, overweight, and obesity. Evaluated reproductive characteristics included: age, basal values of follicle-stimulating hormone (FSH), luteinizing hormone (LH), 17-beta-estradiol (E2), thyroid stimulating hormone (TSH), anti-müllerian hormone (AMH), antral-follicle-count (AFC), duration of COS, E2, and progesterone at the last monitoring, TVUS endometrial thickness at the last monitoring before OPU, FOI after OPU. Additionally, the number of meiosis II oocytes retrieved (MII), the total dose of FSH administered, the ratio between MII and total FSH administered, and OSI were registered.

**Results:**

AMH levels were significantly lower in obese patients compared to normal weight and overweight women (1.05 IQR 1.20, 1.58 IQR 2.16, 1.32 IQR 1.38, respectively, *p*-value = 0.032). When looking at the MII/FSH ratio, the normal weight group showed a median value of 3.3 with an IQR of 4.0, the overweight group showed a median value of 2.3 with an IQR of 1.9, and the obese group had a median value of 2.6 with an IQR of 2.8. Those data were statistically significant (*p*-value = 0.049).

**Conclusion:**

These results emphasize the importance of considering weight status in fertility assessment and treatment planning.

## Introduction

Obesity represents a significant concern for global public health, with implications for various aspects of health, including reproductive health. Studies have shown that weight status can influence reproductive characteristics. Obesity has adverse effects on reproduction, including ovulatory and menstrual function, natural fertility rates, fecundity, infertility treatment success rates, infertility treatment safety, and obstetric outcomes (Willett et al., 1995). Obesity is a disease caused by excess body fat. It increases the risk of a range of common conditions, including type 2 diabetes, dyslipidemia, hypertension, coronary heart disease, gallstones, endometrial and postmenopausal breast cancer, stroke, osteoarthritis, and infertility. (Willett et al., 1995; Colditz et al., 1995; Huang et al., 1998; Maclure et al., 1989; Huang et al., 1997; Lauby-Secretan et al., 2016; Walker et al., 1996; Rich-Edwards et al., 1994). Body fat is challenging to measure directly and is often estimated by calculating Body Mass Index (BMI). Adult weight gain is a significant risk factor for chronic disease and reduced fecundity (Gaskins et al., 2015). Obesity can impair reproduction in both women and men, leading to infertility in couples trying to conceive and subsequent complications in pregnancy (ACOG Practice Bulletin, 2015; Craig et al., 2017; Broughton and Moley, 2017; Stein and Leventhal, 1935). Most studies report a prevalence of menstrual cycle irregularities in women with obesity of 30%–36% (Rogers and Mitchell, 1952; Alvarez-Blasco et al., 2006; Escobar-Morreale et al., 2017; Polotsky et al., 2010; Castillo-Martinez et al., 2003; Hartz et al., 1979). Ovulatory dysfunction is more common in women with obesity (Stein and Leventhal, 1935; Rogers and Mitchell, 1952; Legro et al., 2012; Bhandari et al., 2016; Grodstein et al., 1994). Body fat distribution is also crucial because anovulatory women have a greater waist circumference and more abdominal fat than ovulatory women of similar BMI (Kuchenbecker et al., 2010). Understanding the relationship between weight status and reproductive parameters is essential for optimizing fertility treatment strategies and improving patient outcomes. The oocyte retrieval may be evaluated through the ratios: follicle-to-oocyte index (FOI) – intended as the ratio between the number of retrieved oocytes and the AFC at the beginning of COS; follicular output rate (FORT) – defined as the ratio between the count of pre-ovulatory follicles obtained after COS and the pre-stimulation pool of small antral follicles; and ovarian sensitivity index (OSI) – which represents the ratio between the count of retrieved oocytes and the total dose of FSH administered (Alviggi et al., 2018; Grynberg and Labrosse, 2019; Yadav et al., 2019). Primary aim of the present study is to determine whether there is a relationship between BMI and mature oocytes retrieval after Ovum Pick-Up (OPU). Secondarily, we intended to associate BMI to reproductive characteristics, including hormonal values, antral follicle count (AFC), and endometrial assessment at transvaginal ultrasound (TVUS) evaluation during COS.

## Methods

The retrospective monocenter cohort study collected data from 191 patients undergoing ART at the Infertility Center of the University of Campania Luigi Vanvitelli. COS was based on GnRH antagonist protocol. Patients were divided into three groups based on their weight status: normal weight (BMI 18.5–24.99 kg/m^2^), overweight (BMI 25–29.99 kg/m^2^), and obesity (BMI >30 kg/m^2^). Evaluated reproductive characteristics included: age, basal values of follicle-stimulating hormone (FSH), luteinizing hormone (LH), 17-beta-estradiol (E2), thyroid stimulating hormone (TSH), anti-müllerian hormone (AMH), antral follicle count (AFC), duration of COS, E2 and progesterone at the last monitoring, TVUS endometrial thickness at the last monitoring before OPU, FOI after OPU. Additionally, the number of meiosis II oocytes retrieved (MII), the total dose of FSH administered, the ratio between MII and total FSH administered, and OSI were registered.

## Stimulation protocol

Patients underwent ovarian stimulation based on a flexible GnRH antagonist protocol. Stimulation was carried out identically in the two groups, with 150-to-300 IU of recombinant FSH from day 2 or 3 of their menstrual cycle. The dosage of gonadotrophins for each patient was adapted on their clinical characteristics, with proper modifications during stimulation. GnRH antagonist was injected daily to prevent premature ovulation from the day the leading follicle reached 14 mm diameter until the hCG injection. Ovulation induction was monitored through TVUS and hormonal assessment every 2 days. When at least two follicles had reached 18 mm diameter, a single intramuscular bolus of 10.000 IU of hCG (Gonasi HP 5000; IBSA, Rome, Italy) was administered. Follicular punction was performed 34-to-36 h following the hCG injection.

### Laboratory procedures

Oocytes were cultured in Petri dishes in IVF-20 (Vitrolife, Göteborg, Sweden) at 37 °C in a humidified 5% carbon dioxide/95% ambient air. The semen was processed with 80% Percoll (Sigma Chemical Company, St. Louis, MO) discontinuous gradient centrifugation at 800 *g* for 15 min.

### Statistical analysis

The sample was stratified according to the three BMI classes and analyzed according to the outcome of MII.

The nominal variables were expressed as absolute frequency and percentages. Continuous variables were expressed as median and interquartile range and compared using the Kruskal-Wallis test due to the nonparametric distribution (Kruskal and Wallis, 1952).

The *null* hypothesis of our study was that there was no difference in the mean values of MII between the 3 BMI groups of patients. (H0: μ1 = μ2 = μ3). The distribution of the continuous variables for the individual parameters of the reference outcome was graphed in boxplots.

The weights of the individual values on the continous dependent variable MII were calculated as a linear regression (Montgomery et al., 2012). The significance of the model used was assessed using the maximum likelihood method (Casella and Berger, 2002).

The statistical significance level was set at 0.05, and all statistical investigations were performed using R software and R Studio vers. 2023.12.1 + 402.

## Results

191 patients were enrolled between January 2023 and January 2024.122 patients belonged to the Normal weight category, 40 were overweight, and 29 were obese. Patients’ age was similar between normal weight and overweight individuals, whereas obese women were significantly older on average (BMI 36.0 IQR 7.8, 36.0 IQR 8.0, 39.0 IQR 3.0, respectively, *p*-value = 0.015). There were no differences in FSH, LH, basal E2, and TSH among the three weight categories. AMH levels were significantly lower in obese patients than normal weight and overweight women (1.05 IQR 1.20, 1.58 IQR 2.16, 1.32 IQR 1.38, respectively, *p*-value = 0.032). The AFC at TVUS evaluation did not differ significantly among weight categories (*p*-value = 0.073), although obese patients showed lower AFC values (5.5 IQR 7.0) compared to normal weight (8.0 IQR 8.0) and overweight (8.0 IQR 6.5). In the context of COS, the duration of gonadotrophins administration, progesterone, and endometrial thickness at last monitoring and FOI did not present differences among the weight categories. E2 at last monitoring was higher in normal-weight patients, compared to overweight and obese women, even though those findings were not statistically significant (893 vs. 779 vs. 809, *p*-value = 0.2). Those findings are summarized in Table 1.

**TABLE 1:** Patients’ characteristics.

Characteristic	Normal weight, N = 122^a^	Overweight, N = 40^a^	Obesity, N = 29^a^	*p*-value^b^
Age	36.0, (7.8)	36.0, (8.0)	39.0, (3.0)	**0.015**
FSH	7.53, (4.50)	8.30, (4.10)	6.70, (3.72)	0.5
LH	5.55, (3.18)	5.00, (2.90)	4.70, (2.80)	0.077
E2	46, (31)	37, (28)	46, (45)	0.3
TSH	1.80, (0.80)	1.80, (1.42)	1.55, (0.63)	0.2
AMH	1.58, (2.16)	1.32, (1.38)	1.05, (1.20)	**0.032**
AFC	8.0, (8.0)	8.0, (6.5)	5.5, (7.0)	0.073
Day of Treatment	9.00, (2.00)	10.00, (2.00)	10.00, (2.00)	0.3
E2 in treatment	893, (812)	779, (508)	809, (642)	0.2
Progesterone	0.50, (0.44)	0.50, (0.49)	0.35, (0.31)	0.2
Endometrial Thickness	9.00, (2.00)	10.00, (3.00)	10.00, (1.50)	0.2
FOI	0.80, (0.53)	0.77, (0.43)	0.93, (0.70)	0.5

^a^ Median, (IQR).

^b^ Kruskal-Wallis rank sum test.

**FIGURE 1:**
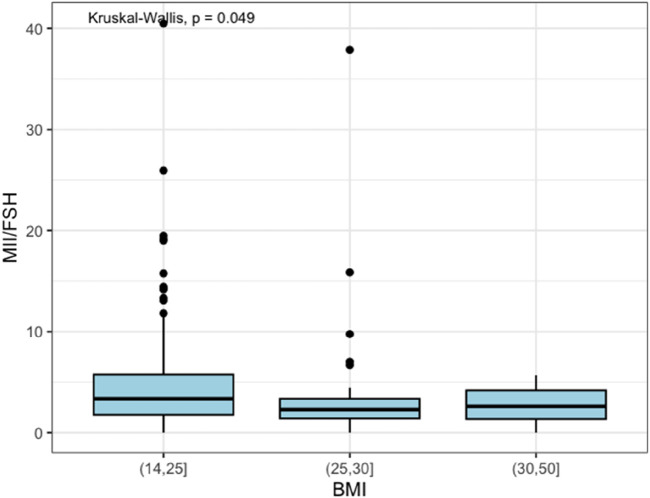
MII Box plot.

Regarding the number of MII oocytes retrieved at OPU, it was observed that the normal weight group had a median value of 5.0 MII oocytes retrieved with an IQR of 3.3. In contrast, both overweight and obese groups had a median value of 4.0 MII oocytes retrieved with an IQR of 4.0. However, those findings failed to reach statistical significance (*p*-value = 0.067). The overweight category required a higher total dose of FSH administered during COS, compared to normal weight and obese categories, with no significant difference (*p*-value = 0.4). However, when looking at the MII/FSH ratio, the normal weight group showed a median value of 3.3 with an IQR of 4.0, the overweight group showed a median value of 2.3 with an IQR of 1.9, and the obese group had a median value of 2.6 with an IQR of 2.8. As represented in Figure 1, those data were statistically significant (*p*-value = 0.049).

**FIGURE 2:**
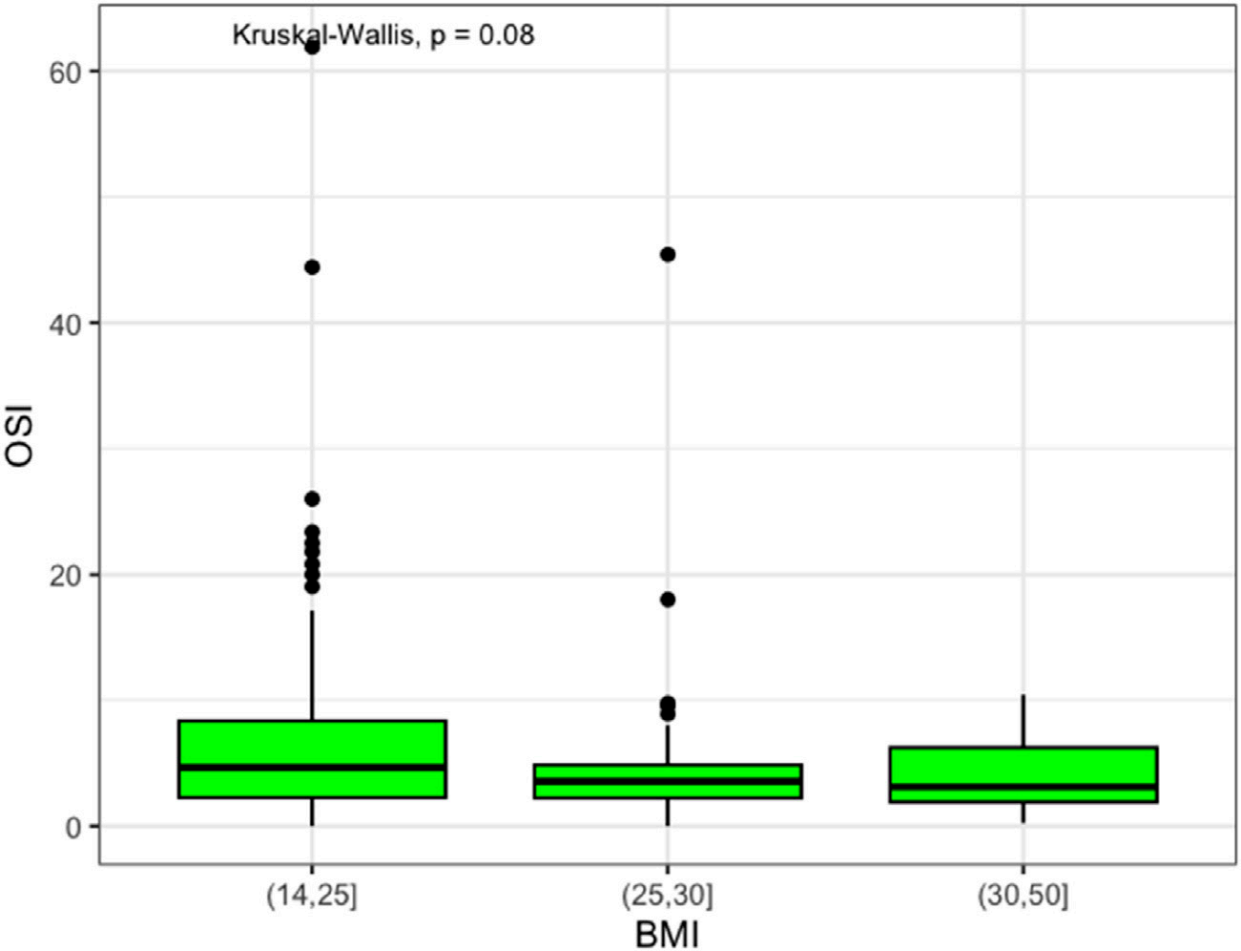
OSI Box plot.

Moreover, ovarian responsiveness to gonadotrophins did not present significant differences among the weight groups, even though normal weight patients showed an OSI of 4.7 (IQR 6.1), which was higher than overweight and obese patients (3.5, IQR 2.6, and 3.1, IQR 4.3, respectively), as demonstrated in Figure 2.

Data are presented in Table 2.

**TABLE 2:** Outcomes.

Characteristic	Normal weight, N = 122^a^	Overweight, N = 40^a^	Obesity, N = 29^a^	*p*-value^b^
MII	5.0, (3.3)	4.0, (4.0)	4.0, (4.0)	0.067
Total FSH	1,458, (895)	1,723, (1,140)	1,440, (1,170)	0.4
MII/FSH	3.3, (4.0)	2.3, (1.9)	2.6, (2.8)	**0.049**
OSI	4.7, (6.1)	3.5, (2.6)	3.1, (4.3)	0.080

^a^ Median, (IQR).

^b^ Kruskal-Wallis Rank sum test.

**FIGURE 3:**
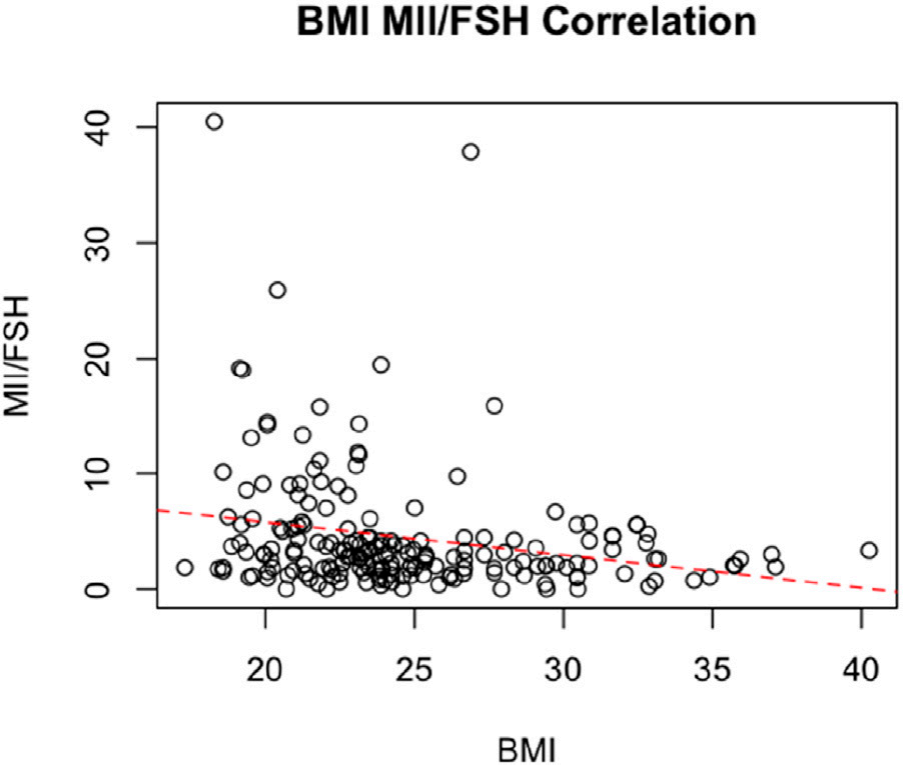
Linear Regression correlating BMI and MII/FSH.

Finally, we performed a linear regression correlating BMI, and MII/FSH., the analysis showed an inverse correlation between the two variables, with a statistically significant negative coefficient of - 0.28 (SE, 0.086; Adjusted R-squared: 0.05; *p* = 0.001) (Figure 3).

**FIGURE 4:**
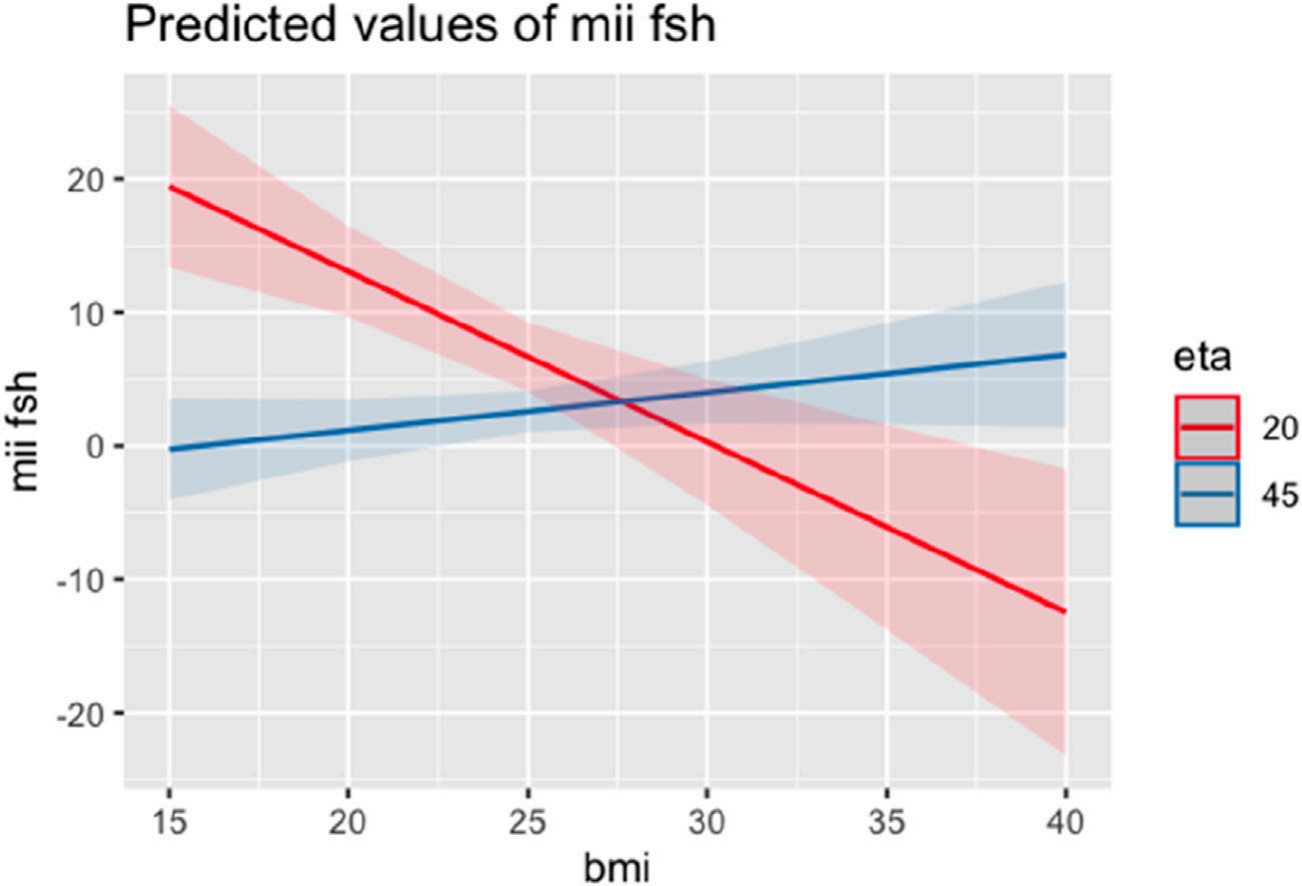
Regression model correlating BMI and MII/FSH including age.

A second regression model included age among the independent variables to avoid the confounder. Also in this second model, BMI maintained an inverse correlation with MII/FSH at the same age from age (−2.53 SE 0.64; Adjusted R-squared: 0.13; *p* < 0.001) (Figure 4).

## Discussion

The results highlight the association between weight status and reproductive characteristics, underscoring the potential impact of obesity on ovarian reserve and fertility outcomes. Those findings suggest that obesity may negatively influence oocyte maturation, as indicated by the lower MII/FSH ratio in obese patients. The observed differences in AMH and AFC levels suggest that obesity is potentially associated with reduced reproductive potential. In particular, median AMH levels were significantly lower in obese patients than normal weight and overweight women (1.05 IQR 1.20, 1.58 IQR 2.16, 1.32 IQR 1.38, respectively, *p*-value = 0.032). In parallel, median AMH values in the normal weight and in the overweight group proportionally decreased. Furthermore, OSI may be influenced by body weight. It was observed that OSI proportionally decreased along with the BMI increase. That corresponds to the expected results because the dose of FSH administered is diluted in a greater volume of distribution. That might depend on a molecular pathway first tested in mice based on the reduction of body fat following the blockage of FSH (Liu et al., 2017). Here is the importance of appropriate counseling with overweight and obese patients, highlighting the necessity of losing weight before COS. A particular finding, which may seem in contrast with other results, lies in the MII/BMI ratio, which was higher in normal weight women. However, it was inferior in overweight patients and obese subjects. The significance of that result has not yet been entirely explained. It would be helpful to assess the outcomes of embryo-transfers in those categories of patients, as well as Live Birth Rates (LBR), to estimate the evolutionary process of IVF. Moreover, most overweight women–who struggle to lose weight–may denote a misleading condition of Polycystic Ovary Syndrome (PCOS), characterized by a different serum and follicular fluid metabolome, affecting oocyte quality (Hood et al., 2023; Kotlyar and Seifer, 2023). In those cases of PCOS subtending insulin resistance, it would be optimal to administer patients with metformin during the IVF protocol. In addition, linear regression proved with robust statistical significance an indirect correlation between BMI and the ratio of metaphase II oocytes to FSH used. This correlation remains even net of age, which could have been a confounder given the higher mean age in the obese patient group. This means that body fat affects the success of the reproductive technique not only by affecting the peripheral distribution of gonadotropins and thus their dosage but also by negatively impacting oocyte quality. How this happens is not entirely clear, but it could be related to the chronic metainflammatory state that accompanies obesity and leads to all its most common complications. Oocyte quality in obese women is mainly affected by the pro-inflammatory status caused by body fat. In a case-control study of 597 women with anovulatory primary infertility compared with 1,695 primiparous controls, the crude and adjusted (for age and exercise) relative risks of primary anovulatory infertility were 3.1 (95% confidence interval [CI], 2.2–4.4) and 2.4 (95% CI, 1.7–3.3) above a BMI of 27 kg/m^2^ (Broughton and Moley, 2017). Another study supported this conclusion by demonstrating that abdominal body fat was more predictive of ovulatory dysfunction than total body fat (Zaadstra et al., 1993). Several studies have investigated the association between obesity and oocyte and resultant embryo quality. Women with obesity undergoing *in vitro* Fertilization (IVF) have an altered follicular environment with higher levels of insulin, markers of inflammation, and elevated levels of free fatty acids, which were correlated with abnormal cumulus-oocyte complexes (Robker et al., 2009; Jungheim et al., 2011; Leary et al., 2015; Caprio et al., 2015). A higher number of oocytes from women who are overweight or obese is immature compared to those from normal-weight patients. In 2016, two large retrospective studies using national data from the Centers for Disease Control and Prevention’s National ART Surveillance System and the SART Clinic Outcome Reporting System database analyzed the relationship between BMI and IVF outcomes. Both studies demonstrate a decrease in pregnancy rate and LBR with increasing BMI (Leary et al., 2015; Marquard et al., 2011; Provost et al., 2016; Kawwass et al., 2016). However, the age-related decline in fertility has a greater impact than BMI on LBR at older ages, suggesting that taking time to lose weight before IVF may be detrimental for older women with overweight or obesity (Goldman et al., 2019; Yao et al., 2024). It would also be interesting to determine the embryonic assessment of the resulting embryos or blastocysts, which has already been demonstrated at a molecular level (Morimoto et al., 2024). Another limitation of the study is that it did not exclude polymorphisms, which are associated with genotypical differences in signaling and receptors (Greco et al., 2023).

The strength of the study is the high number of COS cycles, the single-center design with the same laboratory protocols applied throughout the entire enrollment period, and the same biologists categorizing the oocytes retrieved at OPUs to minimize the interobserver variability. The weakness of the study lies in its retrospective design, which impeded the correlation of collected data with other parameters, such as–for example,–inflammatory indexes. Another limitation of the study is the lack of a sub-analysis of Polycystic Ovary Syndrome (PCOS) patients, which is, normally, negatively associated with the ovarian reserve indicators (Kloos et al., 2024) Those results underscore the importance of weight management in the treatment of infertility, as addressing obesity could improve reproductive outcomes and enhance the success of assisted reproductive procedures.

## Conclusion

Weight status appears to influence various reproductive characteristics in women undergoing fertility treatments. Obesity is associated with lower levels of AMH and AFC, suggesting potential implications for ovarian reserve and fertility outcomes. Considering weight status in fertility evaluation and treatment planning is essential to optimize outcomes and improve patient care in assisted reproductive settings. Further research is needed to clarify the underlying mechanisms of the association between obesity and reproductive function and to develop targeted interventions to mitigate its negative effects on fertility also in clinical practice.

## Data Availability

The original contributions presented in the study are included in the article/Supplementary Material, further inquiries can be directed to the corresponding author.
